# Internet-Based Psychotherapy Intervention for Depression Among Older Adults Receiving Home Care: Qualitative Study of Participants’ Experiences

**DOI:** 10.2196/27630

**Published:** 2021-11-22

**Authors:** Xiaoling Xiang, Jay Kayser, Yihang Sun, Joseph Himle

**Affiliations:** 1 School of Social Work University of Michigan Ann Arbor, MI United States

**Keywords:** internet-based cognitive behavioral therapy, homebound older adults, home care, direct care workers, depression, qualitative study

## Abstract

**Background:**

Depression is common among homebound older adults. Internet-based cognitive behavioral therapy (iCBT) is a promising but understudied approach for treating depression among older adults with disabilities.

**Objective:**

This study aims to understand the experiences of homebound older adults who participated in a pilot feasibility trial of an iCBT for depression.

**Methods:**

The participants included 21 homebound older adults who participated in a generic iCBT program that was not specifically designed for older adults and 8 home care workers who assisted in the iCBT program. Informants completed semistructured individual interviews, which were transcribed verbatim and analyzed using methods informed by grounded theory. A hierarchical code structure of themes and subthemes was developed after an iterative process of constant comparisons and questionings of the initial codes. The data analysis was conducted by using dedoose, a web app for mixed methods research.

**Results:**

Three themes and various subthemes emerged related to participants’ experience of the iCBT intervention, as follows: intervention impact, which involved subthemes related to participants’ perceived impact of the intervention; challenges and difficulties, which involved subthemes on the challenges and difficulties that participants experienced in the intervention; and facilitators, which involved subthemes on the factors that facilitated intervention use and engagement.

**Conclusions:**

iCBT is a promising intervention for homebound older adults experiencing depression. Home care workers reported improved relationships with their clients and that the program did not add a burden to their duties. Future programs should involve accessible technical features and age-adapted content to improve user experience, uptake, and adherence.

**Trial Registration:**

ClinicalTrials.gov NCT04267289; https://clinicaltrials.gov/ct2/show/NCT04267289

## Introduction

### Background

Homebound older adults receive services and support from home care workers (HCWs) to help them maintain community living [1]. Up to half of these older adults experience clinically significant depressive symptoms, and 14% of them meet the diagnostic criteria for current major depression [2]. Untreated and undertreated depression, even when the symptoms are mild, can lead to various adverse health events and decrease life expectancy [3,4]. Psychotherapy interventions based on the principles of cognitive behavioral therapy (CBT) are an evidence-based approach for treating depression in older adults [5]. The side effects of antidepressants, compounded by comorbid conditions and potential interactions with other medications, make psychotherapy a safe alternative [6]. However, office-based psychotherapy treatment is often out of reach for homebound older adults owing to access barriers, such as stigma, provider shortage, cost, and transportation [7].

In recent years, internet-based psychotherapy has received increased attention as a promising alternative to face-to-face treatment. Internet-based CBT (iCBT) delivers prerecorded CBT lessons via dedicated websites or apps. It allows patients to engage in therapy on demand and provides a nonstigmatizing and accessible treatment option. A recent systematic review suggested that iCBT is as effective as face-to-face CBT for depression in mixed-age samples [8]. An emerging body of research also suggests that iCBT is acceptable and potentially effective in reducing depressive symptoms in later life [9]. In addition, a recent randomized controlled trial reported no significant age-related differences in iCBT effectiveness [10].

Most iCBT programs are self-directed, with or without therapist support. When available, therapist support is typically no more than 10 minutes per week per patient [11]. With proper clinical supervision, a layperson or peer can also facilitate iCBT uptake in place of a trained therapist [12]. Guided iCBT, whether by a therapist or by a layperson, may be more efficacious than self-guided iCBT because it provides more opportunities for addressing technological challenges and personalized feedback [13].

However, older adults, especially those with chronic conditions or disabilities, are often excluded from iCBT trials [14]. The few studies that have included them frequently required participants to have computer literacy and access to the internet or a computer [9]. As a result, it is unclear whether previous findings can be extended to homebound older adults who tend to be older, more cognitively and physically impaired, and less familiar with technology [15].

To address this knowledge gap, we conducted an open trial that explored the acceptability and preliminary effects of an existing iCBT program in a sample of homebound older adults, with optional support from their regular HCWs [16]. Our quantitative evaluation found that iCBT was acceptable and associated with a significant reduction in depressive symptoms in the study sample, who were overwhelmingly low income and had low computer literacy. Despite these encouraging findings, the overall completion rate was 23%, which is much lower than the 55%-97% rates reported in previous iCBT trials with older adults [9]. Adherence and symptom improvement have a dose-response relationship [17]. The low completion rate is a cause for concern and warrants further investigation.

### Objective

In this study, we focused on qualitative data from interviews to understand participants’ experiences with iCBT. Qualitative research can provide valuable insights into treatment mechanisms, strengths, and weaknesses, enhance understanding of how interventions are delivered, and guide future treatment innovation [18]. Qualitative analysis is particularly needed on this subject, given the minimal research involving homebound older adults in technology-based psychotherapy. We also analyzed interviews with HCWs to understand their roles in iCBT uptake. We discussed the themes that emerged from the qualitative analysis and recommendations for designing internet-based psychotherapy for depression in homebound older adults.

## Methods

### Participants and Procedures

The trial methodology and procedures have been described previously [16]. The study was registered at ClinicalTrials.gov as NCT04267289. All participants provided written informed consent. Briefly, 26 homebound older adults, recruited from community advertisements and referrals, agreed to participate in the study and started the treatment program. To qualify for the study, older adults needed to be ≥60 years, to be able to read and speak English, to have elevated depressive symptoms at screening (≥5 on the Patient Health Questionnaire-9), and to have received home care (ie, paid help from an HCW) for more than 1 month at screening and expect continued care for at least 3 months. The exclusion criteria included current engagement in psychotherapy, current suicidal ideation, a diagnosis of psychotic disorder, cognitive impairment [19], or a terminal illness diagnosis.

Participants had 3 months to use a commercially available iCBT program called Beating the Blues (BTB). We used an American-specific version of BTB, an iCBT program that was developed in the United Kingdom. Randomized controlled trials of BTB support its clinical effectiveness in treating both major and minor depression in both younger and older adults [20-22]. BTB included didactic content (in the form of lessons), in-session practices, and homework assignments designed to impart cognitive (eg, guided discovery, thought records) and behavioral (eg, activity scheduling, problem-solving) skills. The program has 8 sessions, each consisting of 3-5 modules. The program recommends that users complete a module in one sitting position. Most of the modules took study team members, who were able-bodied and computer literate, about 15-20 minutes to complete, although several more extended modules took about 30 minutes.

Users engage in BTB via a secure and dedicated website compatible with different devices (eg, laptop computers, tablets, and mobile phones). Each user’s progress is saved immediately on the server, allowing them to pick up where they left off from either the same or a different device. Device ownership was not a requirement for participation. For participants without a reliable device (19/26, 73%), we provided each with a Samsung tablet (Galaxy Tab A 10.1 with 4G Long-Term Evolution) and free internet access for the program’s duration. We used ManageEngine’s mobile device management software to uniformly configure and manage all the study team’s tablets. Tablet configurations allowed access to only the BTB website. Most drop-down menu options on the home screen were disabled to prevent accidental contact. We also provided a stylus pen to each participant when the touchscreen failed to respond to finger tapping.

The BTB lessons and materials were entirely web-based. We designed a client workbook and printed it out for each participant. The workbook contained general information about the study, unique user credentials for the BTB website, the study team’s contact information, technology troubleshooting frequently asked questions, a list of all BTB modules with extra space for taking notes, and homework printouts.

When possible, older adult participants were paired with one of their current HCWs to assist with the BTB (13/26, 50%). All HCWs completed a mandatory 2-hour, asynchronous, web-based training course that consisted of an introduction to the study, the role of HCWs in the study, light CBT training, psychoeducation, and safety planning around suicidal ideation. Each HCW received a certificate of completion. In cases where consistent assistance from the same HCW was not feasible (13/26, 50%), participants chose to either work on the iCBT program on their own (7/13, 54%) or receive assistance from a research assistant (RA; 6/13, 46%). RAs were students working on their master’s degrees of social work. They did not receive specialized CBT training and were told to limit their assistance to program navigation and technology troubleshooting.

In addition, all the participants received a short tutorial on using the program. For participants using their own devices, we helped them create a bookmark and shortcut to the BTB website. We also set all browser settings to *remember* user credentials, yet another strategy to reduce user frustration related to technology. Furthermore, we conducted brief check-in calls to each participant approximately once a week, each lasting 2-5 minutes, to monitor symptoms and identify issues requiring immediate intervention. Finally, the participants were told to call the study team for technological issues requiring immediate assistance from the study team.

### Data

All participants, including older adults (n=26) and HCWs (n=13), completed a baseline assessment. We conducted a posttest and semistructured qualitative interview with 21 older adults and 8 HCWs. About half of the older adult participants belonged to the HCW-guided group (n=11), and the rest were split between the RA-guided (n=5) and the self-guided (n=5) groups. Table 1 presents the demographic characteristics of the participants. Further details of the original study sample are available elsewhere [16].

Interviews with older adults were conducted at their homes and recorded using a digital voice recorder. Older adults responded to questions that asked about their program experiences, including probing questions regarding their likes, dislikes, difficulties, perceived impact, and HCW involvement, if applicable. HCWs separately participated in interviews over the phone through BlueJeans, a video conferencing software. HCWs shared their program experiences, perceived impact on client-worker relationships and workload, and web-based training experiences. All interview recordings were transcribed verbatim using a third-party transcription service. The duration of the interviews was 25 minutes. These interviews were the primary data sources for this study. Several members of the study team had close interactions with the participants through home visits and check-in calls. These encounters and observations provided rich contextual data, which were not systematically analyzed, but considered in the coding and interpretation of the interview data.

**Table 1:** Demographics of study participants, including homebound older adults and home care workers (N=29).

Sociodemographic characteristics		Older adults (n=21)	Home care workers (n=8)
Age (years), mean (SD)		76 (9.1)	48 (10.4)
**Sex, n (%)**			
	Female	17 (81)	7 (87)
	Male	4 (19)	1 (13)
**Race and ethnicity, n (%)**			
	White, non-Hispanic	16 (76)	4 (50)
	Black or African American, non-Hispanic	4 (19)	2 (25)
	Hispanic or Latinx	1 (5)	1 (13)
	Asian or Pacific Islander, non-Hispanic	0 (0)	1 (13)
**Education, n (%)**			
	High school or less	4 (19)	2 (25)
	Some college	8 (38)	2 (25)
	AA^a^ or BA^b^	4 (19)	4 (50)
	Graduate degree	5 (24)	0 (0)
**Household income (US $), n (%)**			
	0-20,000	14 (67)	3 (38)
	20,001-50,000	6 (29)	3 (38)
	>50,000	1 (5)	2 (25)
**Marital status, n (%)**			
	Married	1 (5)	5 (63)
	Widowed	6 (29)	0 (0)
	Divorced or separated	11 (52)	2 (25)
	Never married	3 (14)	1 (13)
	Lived alone	18 (86)	1 (13)
**Technology use and competency, n (%)**			
	Ever used a computer	12 (57)	—^c^
	Ever used a tablet	6 (29)	—
**Years worked in home care, mean (SD)**		N/A^d^	12.7 (7.7)

^a^AA: Associate of Arts.

^b^BA: Bachelor of Arts.

^c^Not available (ie, not assessed).

^d^N/A: not applicable.

### Data Analysis

The qualitative data were analyzed using grounded theory [23]. Our analytic process involved line-by-line open coding and refinement through an iterative process of constant comparisons and questionings. The codes were examined within the same participant and across different participants to ensure consistency and reduce redundancy. XX performed open coding and worked with YS. to refine and merge codes and create a hierarchical code structure of themes and subthemes. We conducted data analysis using dedoose, a web app for mixed methods research.

## Results

### Overview

**Figure 1:**
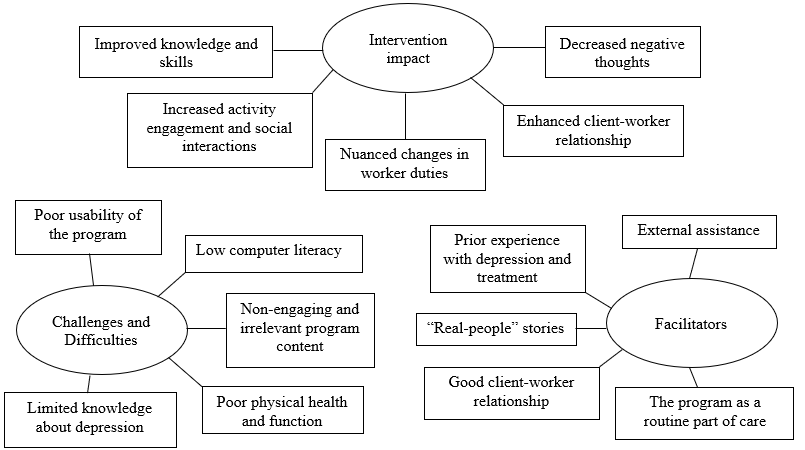
Thematic map depicting themes and subthemes.

During the iterative coding process, we found that older adults’ narratives were congruent with those from HCWs. Therefore, we combined their experiences and reported them on the same thematic map (Figure 1). Themes related to participants’ experience fell under the following three categories: (1) intervention impact, which involved subthemes related to participants’ perceived impact of the intervention, and these subthemes shed light on the intervention mechanisms; (2) challenges and difficulties, which involved subthemes on the challenges and difficulties experienced by participants in the intervention, and these challenges were the likely culprit of low program adherence and completion, and (3) facilitators, which involved subthemes on the factors that facilitated intervention use and engagement.

### Intervention Impact

#### Improved Knowledge and Skills

Participants, including older adults and HCWs, reported improved awareness and knowledge of depression. Several older adults shared that they did not know that they were depressed but after the program they were able to identify their symptoms as typical signs of depression. Participants also reported that they learned skills related to goal setting, problem-solving, and sleep management. HCWs also reported that they applied the skills learned through the program to their own lives and with other clients:

I have got depression. I didn’t even know it was depression, like staying in my room and not going out, or I was invited over for dinner and not going. I would give excuses....It helped me to identify what it is.

71-year-old adult, HCW-guided, completed 5 sessions

Increased activity engagement and social interactions. Another impact of intervention was related to behavioral activation. Older adults reported that they were more active or more motivated to engage in various activities, such as personal care, social events, community activities, and volunteering. They also reported initiating or engaging in more social interactions, which helped to improve their mood:

Most of the time, I’m always up in my room. I don’t really go downstairs. It made me get out to learn how to mingle more with people, and then I found out I kind of like doing that. And then they started a little class, I actually started participating in the Bible study class, and I met more people, more friends. So that, to me, was a big help right there.

71-year-old adult, HCW-guided, completed 8 sessions

#### Decreased Negative Thoughts

Participants reported less negative thinking and more positive thinking. Although some reported having a more positive outlook in general without elaboration, some discussed the process of challenging thoughts in detail. For example:

Helped me to understand...this situation wasn’t my personal failing. Because I’ve heard that throughout my life. It’s still difficult to say, but I’m born out of wedlock...I think I was always, and my mother too, was like, we brought shame to our family....And so, my mother had a nickname for me, which in Spanish it’s like an ugly, old hag, ever since I was a child...you’re programmed to think everything is your fault when it isn’t. So...I would think, “This happened because I’m an ugly, old hag, and I’m corrupt, and I’m contaminated in some way.” But you have to challenge that...I think the program reminded me that this wasn’t my fault....

60-year-old adult, HCW-guided, completed 8 sessions

Enhanced client-worker relationship. A prominent theme in the narratives of participants involved in the HCW-guided group was the closer relationship between the older adult and the HCW. HCWs reported *bonded* or *grew closer* with their clients, which made it easier to understand their needs and to care for them. Older adults’ reports echo those from HCWs:

I think it sort of helped the relationship become more of a friendship because in the course of doing all these exercises, he would tell me about his personal life, so I got to know him better...I think we had a better working relationship. It enhanced his trust in me, and we probably developed a greater respect for each other as people.

HCW

Nuanced changes in worker duties. Most HCWs reported little change in their overall workload and did not perceive the program as an extra burden. For some HCW-client pairs, doing the program gave them activities to do together:

Before the program, we [sometimes] watched TV....This gave us something to do together.

HCW

### Challenges and Difficulties

#### Overview

Poor usability of program. The most robust theme related to challenges and difficulties involved program usability, including web interface and program content. Most participants, including the HCWs, reported experiencing many glitches such as being stuck on a page, receiving error messages, and trouble playing video content at times. Trouble entering text as required was the most frequently reported difficulty. Participants pointed out a few accessibility issues, including small font, low volume of some video content, and small navigation buttons. In addition to the website’s poor usability, many participants also reported some of the lessons that were confusing, repetitive, and challenging to understand. They also overwhelmingly reported that the modules were too long, and they were often unable to finish a module in one sitting:

Well, I got frustrated with it quite a bit. There were several things that I found frustrating. One of them was when you’re supposed to fill out something, and it says to enter in the line, and the line is like, so small that you can hardly see it. And then it’s always giving me this message, “Oops, you forgot something.” And it does that over and over and over....It gets stuck. And you have to go back and start over. It’s a buggy program.

80-year-old adult, self-guided, completed 7 sessions

#### Low Computer Literacy

Another salient challenge was a generally low level of computer literacy among homebound older adults, including those who owned a computer. One participant said, “I’m just computer illiterate, mostly” (72-year-old adult, HCW-guided, completed 1 session).

#### Nonengaging and Irrelevant Program Content

The study participants perceived some aspects of the program as irrelevant to older adults. The case stories in BTB did not depict the common problems and challenges faced by older adults, such as physical and cognitive decline, loneliness and isolation, and loss of independence. As a result, several older adults reported that they could not relate to the characters or had trouble applying the lessons to their life situations. Several participants also thought that the program was not very interesting, which dampened their motivation to continue with the program:

It *wasn’t about older people. Well, they might consider themselves older, but some of them were still working. They’d lost a husband or wife, or something....There just weren’t a whole of a lot of things that pertained to people who were very old.* [94-year-old adult, RA-guided, completed 5 sessions]

Well, it just didn’t create any intense curiosity or, “Where’s this going? How is it going to help?” It was just kind of [laughter] pedestrian or boring.

79-year-old adult, HCW-guided, completed 3 sessions

Poor physical health and function. Participants experienced adverse health events, such as a visit to the emergency room and an overnight hospital stay. Some reported fatigue and malaise, which made it difficult for them to participate in the program:

I’m exhausted. I have difficulty trying to prepare myself for the next day, the next week.

88-year-old adult, HCW-guided, completed 1 session

#### Limited Knowledge of Depression

The final subtheme of challenges is limited knowledge of depression among participants, particularly among those who dropped out within 4 sessions. A few participants identified feelings of anger and shared a lack of motivation, but did not identify them as depression symptoms. Depression denial was common in the baseline clinical interviews. There was also a sentiment that they were *too old to change*. In 1 case, this sentiment made it difficult for the participant to set up a goal, and she dropped out of the program after the first session:

The project itself is an antithesis for me because I can feel anger. I can feel regret. I can feel all kinds of things, but I cannot define depression as a thing. Sometimes, I can feel remorse for something, or I can feel sorry for myself, but I don’t think I do that much.

83-year-old adult, self-guided, completed 4 sessions

### Facilitators

#### External Assistance

The most salient subtheme of facilitators was external assistance, including HCW assistance and support from the research team, such as workbook printouts, stylus pens, and especially help with technology:

Then I’d call [RA]. And then she would fix it; sometimes, she even had to come. But sometimes, she could do it from afar, which I thought was great.

74-year-old adult, HCW-guided, completed 8 sessions

#### Prior Experience with Depression and Treatment

Most participants who completed 7 or 8 sessions spontaneously reported experience with depression and some familiarity with psychotherapy treatments, which helped them better relate to and complete the iCBT lessons:

I mean, I’m familiar with goal setting, I’ve done that before, and how you break down the things into individual chores and all that.

80-year-old adult, self-guided, completed 7 sessions

#### Real People Stories

Many participants liked the characters in the program that told real-life stories. Unknown to the participants, the characters were actors. Nevertheless, it appears that using real-life examples made the program more relatable and engaging. Most participants identified with a character named Rosa, an older woman grieving over losing her husband:

I enjoyed the six or seven people that you tracked through the different modules...I liked listening to what the folks had to say about how they started and the progress through the whole program, and then identify how they were able to use the specific part of the program. I liked it a lot.

74-year-old adult, HCW-guided, completed 8 sessions

#### Good Client-Worker Relationship

Participants in the HCW-assisted group tended to have a better relationship with their HCWs before the study, which improved their experiences with the iCBT program:

Me and my client have actually known each other for a long time already. I already knew a lot about her as it was. So, this was everything that she was telling in the program, I already knew...and about her depression and everything.

HCW

This program is a routine part of care. HCWs who assisted clients with a high adherence to BTB commonly reported making the program a routine part of care. They intentionally adjusted their routine to allocate time for completing the program and integrated homework assignments into the shared activities they did with the clients:

One of the exercises of that week would be exercise while we were out shopping. So, we just incorporated it into our time.

HCW

We were able to set everything around the time that I was scheduled to be with her, and we made that part [the iCBT program] a part of our care.

HCW

In addition to the 3 main themes, we analyzed the roles of HCWs separately and explored why HCW involvement was undesirable for some older adults. We organized the subthemes related to HCW roles based on the efficiency model of support, a model for understanding the provision of human support in the context of behavioral intervention technologies [24]. The premise of this model is that human support increases the adherence and effectiveness of technology-based psychotherapy. The provision of human support should consider failure points and reasons why people might fail to benefit from technology-based psychotherapy. These failure points include usability (ease of use), engagement (motivation), fit (meeting user’s needs), knowledge (how to use a tool within intervention), and implementation (how to apply tools learned into users’ lives). We used the failure point classification to organize the subthemes associated with HCW roles (Table 2).

**Table 2:** Home care worker (HCW) assistance subthemes and representative quotes.

Subthemes	Description	Representative quotes
Usability and fit	Help clients with technology, including the tablet and the program web interface. Specific activities were turning the tablet on and off, logging on, navigating the program (eg, going backward/forward), entering text, reading out loud to clients with vision impairment, solving tech related problems (eg, frozen screen)	“I sat through her segment with her...moving the program along....Actually, I kind of somewhat helped her with the whole program because she needed help to do it. She couldn’t do it by herself*.*” [HCW]
Engagement	Help with engagement through encouraging conservations, nudge and reminders, and hold clients accountable	“She would double-check to make sure that I was doing it....She also encouraged me to do the modules [laughter]....” [74-year-old adult, HCW-guided, completed 8 sessions]
Knowledge	Help clients better understand the lessons, explain and clarify the content, discuss and review lessons, and help them complete the in-session exercises	“If I didn’t quite understand something, he would explain it more fully....A lot of times, I wouldn’t remember, but he could always refer to things.” [72-year-old adult, HCW-guided, completed 8 sessions]
Implementation	Help clients apply the lessons by assisting them complete the homework assignments and continue to practice the skills and techniques during their interactions, even after program completion	“I helped her with her goals of the week. I know some of the goals that she has that were maybe going out into the community or maybe she wanted to get up and do a certain exercise or she wanted to do certain things.” [HCW]
Personal autonomy and privacy	Older adults in the self-guided and research assistant–guided group shared reasons for not wanting to involve their HCW in the internet-based cognitive behavioral therapy program. The strongest theme was the concept of personal autonomy and privacy—the desire to keep somethings private and maintain independence as much as possible	“I don’t want anybody helping me. I am too independent [laughter]”. [80-year-old adult, self-guided, completed 7 sessions]
Competency	Another common reason for not wanting to involve HCWs is concern over HCWs’ competency. There was some concern among several older adults that their HCWs did not possess the professional knowledge to make a difference in their experience	“It’s not like she’s really a professional like you are. But she had some rudimentary exposure with this program, and she knew how and what to help me with. She was a help but still didn’t make the program any more meaningful to me or helpful.” [79-year-old adult, HCW-guided, completed 3 sessions]

## Discussion

### Principal Findings

This study analyzed data from individual interviews with homebound older adults and HCWs who participated in a pilot feasibility trial of iCBT with optional support from lay workers. In our study, homebound older adults tended to be older, more functionally impaired, more socioeconomically disadvantaged, and less tech-savvy than older adults from previous iCBT trials [12]. This study complemented a previous quantitative evaluation of the iCBT program [16] by providing insights into intervention mechanisms, barriers, and facilitators of iCBT uptake in this high-need and hard-to-reach population. Taken together, the qualitative and quantitative findings suggest that iCBT, even a program initially designed for those who are able-bodied and computer literate, is acceptable and can benefit older adults with diverse socioeconomic backgrounds and abilities. Study participants reported that the program improved their knowledge and skills, increased engagement in social activities and interactions, and decreased negative thinking patterns. These changes closely align with the CBT theory and the mechanisms of change in CBT treatment [25]. In addition, participants in the HCW-guided iCBT group, including older adults and HCWs, reported enhanced client-worker relationships with more closeness, trust, and respect. The enhanced relationship made it easier for workers to understand clients’ needs and care for them. We found a consensus from the HCWs that assisting clients with iCBT did not change their workload or work nature. These findings suggest that adding HCW support to iCBT is feasible and can bring benefits beyond symptom reduction.

However, participants’ experience was also marked with difficulties using the program because the interface was not optimized for older adults with diverse abilities and a generally low level of computer literacy among the study population. While learning a new technology can empower and increase a sense of self-efficacy for some older adults [26], technological challenges may cause feelings of disempowerment, aggravating symptoms of stress and anxiety for others [27]. These challenges are common when interacting with a multicomponent complex interface, such as an iCBT program, even among those with moderate to high computer literacy [28].

Overall, the challenges and difficulties experienced by our participants are consistent with those reported by older adults who used MoodTech, an iCBT program with a peer support component specifically designed for older adults with symptoms of depression [28]. Textual data from a pilot trial of MoodTech showed that older adult participants, all of whom were college-educated and owned a computer, reported various difficulties in working with the program interface, particularly those associated with entering and saving text entry. Similarly, text entry was the most frequently reported difficulty and a significant source of frustration with iCBT among homebound older adults in our study, who were less educated and computer savvy than MoodTech users. These difficulties led to confusion, frustration, and self-doubt and fueled the participants’ insecurities in life [28]. Another shared challenge among our study participants and MoodTech users involved the length and pace of iCBT. In both studies, participants reported being overwhelmed by the amount of work involved. The convergence in findings across different iCBT programs (one tailored for older adults and the other being nonage specific) and study populations (one involving college-educated and computer literate older adults and the other involving frail homebound older adults with limited computer literacy) suggests that technological challenges and perceived user burden are common, and that existing iCBT programs need to be further optimized to improve user experience.

A unique barrier to iCBT engagement in our study was the perceived lack of relevance in program content, which contributed to low adherence and engagement. MoodTech was developed explicitly for older adults aged 65 years or above, whereas BTB was not designed for a specific age group. Although general iCBT programs can help treat depression among older adults [29], our qualitative analysis suggests that age adaptations can improve user experience and lead to better adherence, engagement, and clinical effectiveness.

Whether from an HCW or from a member of the study team, receiving external assistance was crucial for a positive user experience and treatment adherence in our study population. External assistance improved the fit between the demands of the program and the capabilities of users. HCWs also frequently provided reminders, encouragement, clarifications, and assistance with homework assignments and skills application. These findings echo those of a qualitative study of patients’ experience of an iCBT program with a face-to-face component, which suggested that in-person meetings compensated for the insufficient tailoring of iCBT to the user’s needs and provided opportunities for individualized discussions about feelings, lessons, and challenges [30].

### Implications for iCBT Design

This study provides several takeaways for designing internet-based psychotherapy programs for older adults with diverse physical, cognitive, and technological abilities. Our findings align with those of other studies that emphasize the importance of a user-friendly program interface [28]. The initial impression of computer interfaces, which is dependent on how easy it is for users to achieve simple tasks, strongly influences users’ attitudes toward their intentions to use a technology [31]. Future interface design must involve careful consideration of accessibility features, including but not limited to button size and spacing, font size, color contrast, and voice-over narration and its volume, and use plain language accessible to those with low health literacy. Moreover, features requiring user input, such as text entry, should be tested with potential end-users to improve usability. Particular attention should be given to text entry, a feature that commonly causes problems for users. Potential remedies include making text entry options and including alternative ways of soliciting user input, such as asking them to write down their responses in a paper workbook accompanying the web-based program. Using voice input and handwriting input features on digital devices is another potential remedy. For example, we showed participants how to enter text using voice typing and handwriting on the tablets. Several participants opted to use voice input, and some went to handwriting. Those who could use either voice or handwriting input commented on how useful it was to bypass finger typing.

One of the most important implications for the future design of iCBT for older adults involves age-appropriate case stories. Although programs without age-appropriate stories can improve depressive symptoms in older adults, age-appropriate case stories may be more effective at engaging users and imparting skills, and as a result, augment treatment effectiveness. Using age-appropriate case stories may be particularly important for individuals with mild cognitive impairment and impaired cognitive flexibility. Examples of age-appropriate case stories, based on participants’ input, include (1) challenges of adaptation to late-onset disabilities, (2) significant life events and transitions (eg, widowhood and change in living arrangements); (3) loneliness and social isolation; (4) family relations (eg, caregiving, multigenerational households, family conflict, and estranged children); (5) financial constraints and housing stability; and (6) early life trauma (eg, childhood sexual abuse). The case-story design should also consider the tremendous diversity of older adults. Old age spans several decades and includes people from several generations. Including stories that reflect this diversity can make the program more relatable and appealing to people from different birth cohorts.

In addition, given that limited knowledge on geriatric depression among older adults is common and can prevent them from engaging in treatment, strong psychoeducation is an essential component in iCBT to educate older adults on depression and address agism and mental illness stigma. When designing psychoeducation, age-related differences in the experience of depressive symptoms should be considered. Older adults are less likely to endorse affective symptoms and identify more strongly with cognitive, somatic, and behavioral symptoms [32]. Those who identify more strongly with nonaffective symptoms also find it more difficult to accept that they may have depression, and as a result, may be less motivated to engage in iCBT. For example, a few participants in our study endorsed anger and irritability but failed to realize that these could be symptoms of depression; none of these participants completed more than 3 sessions. Psychoeducation for older adults, therefore, should educate them about the nonaffective symptoms of depression and commonly held misconceptions.

In terms of program content, excessive repetition may cause confusion, rather than clarification. One way to determine whether repetition might be excessive is to test the program with a few potential end-users with varying cognitive ability levels. Relatedly, shorter individual modules spanning several months may reduce user fatigue and improve adherence.

Beyond program design, external assistance may be required for older adults with moderate to severe impairments in vision, hearing, cognition, and manual dexterity and is desirable for those with limited computer literacy. Our findings suggest that having laypersons as external support is feasible and may improve user experience by resolving technological challenges and clarifying program content.

### Limitations

This study was limited to a small sample size for each of the 3 treatment subgroups, making it more difficult to compare participants’ experiences across subgroups. Moreover, older adult interviewees were primarily female, non-Hispanic White people, and nonmarried individuals. Older men, ethnic minorities, particularly Hispanics and Asians, and married individuals were underrepresented. In addition, our findings were based primarily on individual interviews with study participants. Other data sources, such as text entry, notes in the client workbook, and in vivo observations, were unavailable, but could have provided additional insights to inform future interventions.

### Conclusions

iCBT is a promising intervention approach for reducing the burden of depression among homebound older adults. Participants reported decreased negative thoughts and improved knowledge and skills, consistent with the goals of the program. Those with HCWs also reported enhanced client-worker relationships. Of note, HCWs did not find that adding the program to their regular duties increased their burden, suggesting that adding HCW support to self-guided iCBT is feasible. However, homebound older adults, especially those who started iCBT without external assistance, had many difficulties related to poor usability of the program, nonengaging content, physical limitations, and low computer literacy. These findings suggest that external assistance may be an essential component of iCBT for older adults with diverse abilities. Future research should further investigate sources of support already present in older adults’ environments (eg, family caregivers, HCWs, or a nonhuman assistant like Amazon Alexa) and compare the added benefits of support from different sources on iCBT completion and effectiveness. Our study findings also suggest that adaptations to existing iCBT programs are needed to improve user experience, uptake, and adherence. Future work should further investigate the themes and user preferences exposed through our qualitative work to provide a more detailed articulation of desirable adaptations to program features and session content.

## Abbreviations

BTB: Beating the Blues
CBT: cognitive behavioral therapy
HCW: home care worker
iCBT: internet-based cognitive behavioral therapy
RA: research assistant
